# Supplementary material for: Deciphering the neural signature of human cardiovascular regulation
Source: eLife. 2020 Jul 28;9:e55316. doi: 10.7554/eLife.55316 (PMC7386911; doi:10.7554/eLife.55316)
Supplement: Supplementary file 1. [file elife-55316-supp1.docx]

| **Nucleus** | **Abbr.** | **Mentions in reviews on central cardiovascular control** | | | | |
| --- | --- | --- | --- | --- | --- | --- |
|  |  | Loewy  1981 | Benarroch  1993 | Dampney  1994 | Coote  2004 | Saper  et al. 2015 |
| Paraventricular nucleus | PVN | **+** | **+** | **+** | **+** | **+** |
| Lateral hypothalamic area | LH |  | **+** | **+** | **+** | **+** |
| Arcuate nucleus = Infundibulum = Retrochiasmatic Area | Arc |  | **+** |  | **+** | **+** |
| Dorsomedial hypothalamic nucleus | DMH |  | **+** |  | **+** | **+** |
| Median preoptic nucleus = Preoptic area | MPO | **+** |  |  | **+** | **+** |
| Supraoptic nucleus | SO | **+** |  | **+** |  |  |
| Perifornical area / region / nucleus = Hypothalamic defense area | PeF |  | **+** | **+** |  |  |
| Posterior hypothalamic area | PH |  |  | **+** | **+** |  |
| Anteroventral third ventricle region = Anteroventrolateral region of 3rd ventricle = Anteroventral Periventricular Nucleus | AV3V |  |  | **+** |  | **+** |
| Subfornical organ | SFO | **+** |  |  |  |  |
| Vascular Organ of the Lamina Terminalis | OVLT |  |  |  |  | **+** |
| Suprachiasmatic Nucleus | SCh |  |  |  |  | **+** |
| Supramammillary Nucleus = Retromammillary area | SuM |  |  |  |  | **+** |
| Tuberomammillary Nucleus | TM |  |  |  |  | **+** |
