# Supplementary material for: Deciphering the neural signature of human cardiovascular regulation
Source: eLife. 2020 Jul 28;9:e55316. doi: 10.7554/eLife.55316 (PMC7386911; doi:10.7554/eLife.55316)
Supplement: Supplementary file 2. [file elife-55316-supp2.docx]

| **Nucleus** | **Abbr.** | **Mentions in reviews on central cardiovascular control** | | | |
| --- | --- | --- | --- | --- | --- |
|  |  | Loewy  1981 | Benarroch  1993 | Dampney  1994 | Saper  et al. 2015 |
| Nucleus of the solitary tract (incl. A2/C2 region) | NTS | **+** | **+** | **+** | **+** |
| Rostral ventrolateral medulla (incl. C1 region) = Rostral ventrolateral reticular nucleus = Chemosensitive region of the ventral medulla | RVLM | **+** | **+** | **+** | **+** |
| Caudal ventrolateral medulla (incl. A1 region) | CVLM | **+** | **+** | **+** | **+** |
| Nucleus ambiguus | NA | **+** | **+** | **+** | **+** |
| Medullary raphe nuclei = Caudal raphe nuclei = Nucleus raphe obscurus and Nucleus raphe pallidus | ROb, RPa | **+** | **+** | **+** | **+** |
| Rostral ventromedial medulla = parapyramidal region = Nucleus interfascicularis hypoglossi = Paraolivary nucleus | RVM | **+** |  | **+** | **+** |
| Area postrema | AP |  | **+** | **+** | **+** |
| Dorsal motor nucleus of the vagal nerve | DMN | **+** |  | **+** |  |
| Intermediate ventrolateral medulla | IVLM |  |  | **+** |  |
| Retrotrapezoid nucleus | RTN |  |  |  | **+** |
| Nucleus reticularis gigantocellularis, pars α | GiA | **+** |  |  |  |
| Medullary lateral tegmental field | LTF |  |  | **+** |  |
