# Supplementary material for: Deciphering the neural signature of human cardiovascular regulation
Source: eLife. 2020 Jul 28;9:e55316. doi: 10.7554/eLife.55316 (PMC7386911; doi:10.7554/eLife.55316)
Supplement: Supplementary file 3. [file elife-55316-supp3.docx]

| **IC** | **Maximum [mm]** | | | **Center of gravity [mm]** | | | **Nucleus** | **Hypothalamic Zone** |
| --- | --- | --- | --- | --- | --- | --- | --- | --- |
|  | **x** | **y** | **z** | **x** | **y** | **z** |  |  |
| 1 | 2 | 3 | -10 | 2 | 2 | -9 | Medial preoptic ncl. | periventricular |
| 2 | 5 | 0 | -11 | 4 | 0 | -11 | Lateral hyp. area, anterior hyp. area | lateral |
| 3 | 4 | 1 | -14 | 5 | 1 | -14 | Anterior hyp. area | periventricular |
| 4 | 3 | 0 | -16 | 2 | -1 | -17 | Supraoptic commissure, arcuate ncl. | periventricular |
| 5 | 2 | 1 | -14 | 1 | 1 | -13 | Anterior hyp. area, paraventricular hyp. ncl. | periventricular |
| 6 | 1 | 0 | -21 | 0 | 0 | -20 | Arcuate ncl. | periventricular |
| 7 | -2 | 1 | -9 | -2 | 1 | -9 | Medial preoptic ncl. | periventricular |
| 8 | -6 | 0 | -11 | -5 | 0 | -11 | Lateral hyp. area | lateral |
| 9 | 9 | -1 | -13 | 10 | -1 | -12 | Lateral hyp. area, supraoptic ncl. | lateral |
| 10 | 7 | -2 | -11 | 6 | -2 | -11 | Anterior hyp. area, lateral hyp. area, | periventricular |
| 11 | 2 | -1 | -7 | 2 | -1 | -7 | Paraventricular hyp. ncl. | periventricular |
| 12 | -2 | 0 | -16 | -2 | -1 | -17 | Supraoptic commissure, arcuate ncl. | periventricular |
| 13 | -2 | -1 | -13 | -2 | -1 | -12 | Anterior hyp. area | periventricular |
| 14 | -4 | -1 | -14 | -5 | -2 | -14 | Anterior hyp. area, supraoptic commissure | periventricular |
| 15 | -7 | 0 | -13 | -8 | 0 | -13 | Lateral hyp. area | lateral |
| 16 | 15 | -4 | -11 | 14 | -4 | -11 | Lateral hyp. area, supraoptic ncl. | lateral |
| 17 | 7 | -4 | -13 | 6 | -4 | -13 | Lateral tuberal ncl., perifornical ncl., lateral hyp. area, tuberomamillary ncl. | lateral |
| 18 | 4 | -4 | -8 | 4 | -4 | -8 | Perifornical ncl., fornix | lateral |
| 19 | 3 | -3 | -13 | 2 | -3 | -14 | Ventromedial hyp. ncl. | medial |
| 20 | 2 | -4 | -8 | 2 | -5 | -8 | Dorsomedial hyp ncl.., 3rd ventricle | medial |
| 21 | -2 | -4 | -8 | -3 | -3 | -9 | Dorsomedial hyp. ncl., perifornical ncl., Fornix | medial |
| 22 | -7 | -3 | -11 | -7 | -3 | -12 | Lateral hyp. area | lateral |
| 23 | -11 | -3 | -11 | -12 | -3 | -12 | Lateral hyp. area, supraoptic ncl. | lateral |
| 24 | 9 | -5 | -12 | 9 | -4 | -11 | Lateral hyp. area | lateral |
| 25 | 7 | -6 | -11 | 6 | -6 | -11 | Pallidohyp. ncl., lateral hyp. area | lateral |
| 26 | 4 | -5 | -12 | 4 | -5 | -11 | Fornix, perifornical ncl. | medial |
| 27 | 2 | -5 | -10 | 2 | -4 | -11 | Dorsomedial hyp. ncl. | medial |
| 28 | 2 | -5 | -5 | 2 | -4 | -6 | Posterior hyp. area, paraventricular hyp. ncl. | medial |
| 29 | -2 | -5 | -13 | -3 | -5 | -13 | Perifornical ncl. | medial |
| 30 | -5 | -6 | -10 | -4 | -5 | -10 | Lateral hyp. area | lateral |
| 31 | 4 | -7 | -13 | 4 | -7 | -13 | Medial mamillary ncl. | medial |
| 32 | 4 | -8 | -7 | 4 | -8 | -8 | Posterior hyp. area, lateral hyp. area | medial |
| 33 | 2 | -8 | -5 | 2 | -7 | -6 | Posterior hyp. area, 3rd ventricle | medial |
| 34 | 1 | -7 | -12 | 1 | -6 | -12 | Supramamillary ncl. | medial |
| 35 | -1 | -7 | -11 | -2 | -6 | -10 | Supramamillary ncl., posterior hyp. area | medial |
| 36 | -5 | -7 | -12 | -5 | -6 | -13 | Lateral hyp. area | lateral |
| 37 | 4 | -10 | -10 | 4 | -9 | -11 | Lateral hyp. area | lateral |
| 38 | 2 | -9 | -16 | 1 | -9 | -16 | Medial mamillary ncl. | medial |
| 39 | 1 | -9 | -10 | 1 | -8 | -9 | Posterior hyp. area, 3rd ventricle | medial |
| 40 | 1 | -9 | -12 | 1 | -9 | -12 | Medial mamillary ncl. | medial |
| 41 | -2 | -9 | -8 | -3 | -8 | -8 | Posterior hyp. area | medial |
| 42 | -2 | -9 | -14 | -2 | -8 | -14 | Medial mamillary ncl. | medial |
| 43 | -2 | -10 | -11 | -3 | -9 | -11 | Lateral hyp. area, retromamillary commissure | lateral |
