# Supplementary material for: Deciphering the neural signature of human cardiovascular regulation
Source: eLife. 2020 Jul 28;9:e55316. doi: 10.7554/eLife.55316 (PMC7386911; doi:10.7554/eLife.55316)
Supplement: Supplementary file 4. [file elife-55316-supp4.docx]

| **Fig. 2** | **IC** | **Max. t value** | **Size [mm^3^]** | **Maximum [mm]** | | | **Center of gravity [mm]** | | | **Nucleus** |
| --- | --- | --- | --- | --- | --- | --- | --- | --- | --- | --- |
|  |  |  |  | **x** | **y** | **z** | **x** | **y** | **z** |  |
| a | 9 | 3.98 | 4 | 4 | -43 | -51 | 4.3 | -42.5 | -5 | Ncl. tractus solitarii, dorsal motor ncl., parvicellular reticular ncl. |
| b | 16 | 4.52 | 18 | 15 | -5 | -11 | 13.8 | -4.2 | -11 | Lateral hyp. area, supraoptic ncl. |
|  |  | 4.04 | 2 | -3 | -4 | -12 | -3.5 | -4.0 | -12 | Fornix, Perifornical ncl. |
| c | 23 | 3.82 | 2 | -13 | -3 | -11 | -13.0 | -3.5 | -11 | Lateral hyp. area |
|  |  | -4.00 | 5 | 0 | -1 | -20 | 0.4 | -0.21 | -20.4 | Arcuate ncl. |
| d | 28 | -5.02 | 45 | 3 | -2 | -7 | 2.02 | -3.6 | -6.22 | Paraventricular hyp. ncl. |
|  |  | -4.56 | 5 | -6 | -3 | -13 | -6 | -3.4 | -12.8 | Lateral hyp. area |
|  |  | 4.19 | 54 | 8 | -37 | -53 | 6.82 | -38.1 | -51.8 | Inferior olivary ncl., rostral ventrolateral medulla, intermediate reticular ncl., parvicellular reticular ncl., spinal trigeminal ncl., ncl. ambiguus |
| e | 6 | 4.42 | 36 | 6 | -43 | -50 | 4.52 | -42.4 | -48.6 | Intermediate reticular ncl., ncl. tractus solitarii, dorsal motor ncl., parvicellular reticular ncl., dorsal paragigantocellular ncl., ncl. hypoglossus |
|  |  | 3.98 | 8 | -1 | -45 | -57 | -1.48 | -45.2 | -56.4 | Dorsal motor ncl., ncl. tractus solitarii |
|  |  | 3.63 | 36 | 2 | -42 | -58 | 3.19 | -41.7 | -57.2 | Medullary reticular ncl., ncl. raphe obscurus, intermediate reticular ncl. |
