# Supplementary material for: Deciphering the neural signature of human cardiovascular regulation
Source: eLife. 2020 Jul 28;9:e55316. doi: 10.7554/eLife.55316 (PMC7386911; doi:10.7554/eLife.55316)
Supplement: Supplementary file 5. — Note that the reference scans for unwarping (Reference SE (no SMS; AP and PA)) are the same sequence but with inverted phase encoding direction. Hence, they are summarised in one table here. However, both sequences need to be acquired for estimating the susceptibility induced field. [file elife-55316-supp5.docx]

| **Head Scout** | |
| --- | --- |
| TA: 0:17 PM: REF Voxel size: 1.6×1.6×1.6 mmPAT: 2 Rel. SNR: 1.00 : fl | |
| **Properties** | |
| Prio Recon. | Off |
| Load to viewer | On |
| Inline movie | Off |
| Auto store images | On |
| Load to stamp segments | Off |
| Load to graphic segments | On |
| Auto open inline display | Off |
| Auto close inline display | Off |
| Start measurement without further preparation | On |
| Wait for user to start | Off |
| Start measurements | Single |
| **Routine** | |
| Slab group | 1 |
| Slabs | 1 |
| Dist. factor | 20% |
| Position | L0.0 P20.0 H0.0 mm |
| Orientation | Sagittal |
| Phase enc. dir | A >> P |
| Phase oversampling | 0% |
| Slice oversampling | 0.0% |
| Slices per slab | 128 |
| FoV read | 260 mm |
| FoV phase | 100.0% |
| Slice thickness | 1.6 mm |
| TR | 3.15 ms |
| TE | 1.37 ms |
| Averages | 1 |
| Concatenations | 1 |
| Filter | None |
| Coil elements | HC1-6 |
| **Contrast - general** | |
| TR | 3.15 ms |
| TE | 1.37 ms |
| Flip angle | 8 deg |
| **Contrast - dynamic** | |
| Averages | 1 |
| Averaging mode | Short term |
| Reconstruction | Magnitude |
| Measurements | 1 |
| **Resolution - general** | |
| FoV read | 260 mm |
| FoV phase | 100.0% |
| Slice thickness | 1.6 mm |
| Base resolution | 160 |
| Phase resolution | 100% |
| Slice resolution | 69% |
| Phase partial Fourier | 6/8 |
| Slice partial Fourier | 6/8 |
| Trajectory | Cartesian |
| **Resolution - iPAT** | |
| PAT mode | GRAPPA |
| Accel. factor PE | 2 |
| Ref. lines PE | 24 |
| Accel. factor 3D | 1 |
| Reference scan mode | Integrated |
| **Resolution - filter image** | |
| Image filter | Off |
| Distortion corr. | Off |
| Prescan norm. | Off |
| Unfiltered images | Off |
| Normalize | Off |
| B1 filter | Off |
| **Resolution - filter raw data** | |
| Raw filter | Off |
| Elliptical filter | Off |
| **Geometry - general** | |
| Slab group | 1 |
| Slabs | 1 |
| Dist. factor | 20% |
| Position | L0.0 P20.0 H0.0 mm |
| Orientation | Sagittal |
| Phase enc. dir | A >> P |
| Slice oversampling | 0.0% |
| Slices per slab | 128 |
| FoV read | 260 mm |
| FoV phase | 100.0% |
| Slice thickness | 1.6 mm |
| TR | 3.15 ms |
| Multi-slice mode | Sequential |
| Series | Ascending |
| Concatenations | 1 |
| **Geometry - AutoAlign** | |
| Slab group | 1 |
| Position | L0.0 P20.0 H0.0 mm |
| Orientation | Sagittal |
| Phase enc. dir | A >> P |
| Initial position | Isocentre |
| L | 0.0 mm |
| P | 0.0 mm |
| H | 0.0 mm |
| Initial rotation | 0.0 deg |
| Initial orientation | Transversal |
| **System - Misc** | |
| Positioning mode | REF |
| Table position | H |
| Table position | 0 mm |
| MSMA | S - C - T |
| Sagittal | R >> L |
| Coronal | A >> P |
| Transversal | F >> H |
| Coil Combine Mode | Adaptative Combine |
| Save uncombined | Off |
| Optimisation | Off |
| Coil selection | Off - AutoCoilSelect |
| **System - Adjustment** | |
| B0 shim mode | Tune-Up |
| B1 shim mode | TrueForm |
| Adjust with body coil | Off |
| Confirm freq. adjustment | Off |
| Assume Dominant Fat | Off |
| Assume Silicone | Off |
| Adjustment Tolerance | Auto |
| **System - Adjustment volume** | |
| Position | Isocentre |
| Orientation | Transversal |
| Rotation | 0.00 deg |
| A >> P | 263 mm |
| R >> L | 350 mm |
| F >> H | 350 mm |
| Reset | Off |
| **System - pTx Volume** | |
| B1 shim mode | TrueForm |
| Excitation | Non-sel |
| **System - Tx/Rx** | |
| Frequency 1H | 123.225865 MHz |
| Correction factor | 1 |
| Gain | Low |
| Image Scale Cor. | 1.000 |
| Reset | Off |
| ? Ref. Amplitude 1H | 0.000 V |
| **Physio - PACE** | |
| Resp. control | Off |
| Concatenations | 1 |
| **Inline - General** | |
| Flip angle | 8 deg |
| Measurements | 1 |
| Time to k-space center | 7.5 s |
| **Inline - Inline** | |
| Subtraction | Off |
| Measurements | 1 |
| StdDev | Off |
| Save original images | On |
| **Inline - MIP** | |
| MIP-Sag | Off |
| MIP-Cor | Off |
| MIP-Tra | Off |
| MIP-Time | Off |
| Save original images | On |
| **Inline - Composing** | |
| Distortion corr. | Off |
| **Sequence - Part 1** | |
| Introduction | On |
| Dimension | 3D |
| Asymetric echo | Weak |
| Contrasts | 1 |
| Multi-slice mode | Sequential |
| Bandwidth | 540 Hz/Px |
| **Sequence - Part 2** | |
| RF pulse type | Fast |
| Gradient mode | Normal |
| Excitation | Non-sel |
| RF spoiling | On |
| **Sequence - Assistence** | |
| Mode | Off |

| **Functional GRE EPI** | |
| --- | --- |
| TA:20:42 PM: FIX Voxel size:2.0x2.0x2.0 mmPAT: 6 Rel. SNR:1.00 :epfid | |
| **Properties** | |
| Prio Recon. | Off |
| Load to viewer | On |
| Inline movie | Off |
| Auto store images | On |
| Load to stamp segments | Off |
| Load to graphic segments | Off |
| Auto open inline display | Off |
| Auto close inline display | Off |
| Start measurement without further preparation | Off |
| Wait for user to start | Off |
| Start measurements | Single |
| **Routine** | |
| Slab group | 1 |
| Slabs | 78 |
| Dist. factor | 0% |
| Position | L0.0 A5.4 F4.1 mm |
| Orientation | T > S2.3 > C-1.6 |
| Phase enc. dir | A >> P |
| AutoAlign | Head > Brain |
| Phase oversampling | 0% |
| FoV read | 208 mm |
| FoV phase | 86.5% |
| Slice thickness | 2.0 mm |
| TR | 1230 ms |
| TE | 32.0 ms |
| Averages | 1 |
| Concatenations | 1 |
| Filter | Prescan normalisation |
| Coil elements | HC1-7 |
| **Contrast - general** | |
| TR | 1230 ms |
| TE | 32.0 ms |
| MTC | Off |
| Flip angle | 65 deg |
| Fat suppr. | Fat sat. |
| **Contrast - dynamic** | |
| Averages | 1 |
| Averaging mode | Long term |
| Reconstruction | Magnitude |
| Measurements | 1000 |
| Delay in TR | 0 ms |
| Multiple series | Off |
| **Resolution - general** | |
| FoV read | 208 mm |
| FoV phase | 86.5% |
| Slice thickness | 2.0 mm |
| Base resolution | 104 |
| Phase resolution | 100% |
| Phase partial Fourier | 7/8 |
| Interpolation | Off |
| **Resolution - iPAT** | |
| Acceleration mode | Slice |
| Accel. factor PE | 1 |
| Ref. lines PE | 12 |
| Accel. factor slice | 6 |
| Reference scan mode | EPI/separate |
| **Resolution - filter image** | |
| Image filter | Off |
| Prescan norm. | On |
| **Resolution - filter raw data** | |
| Raw filter | Off |
| Elliptical filter | Off |
| Hamming | Off |
| **Geometry - general** | |
| Slab group | 1 |
| Slabs | 78 |
| Dist. factor | 0% |
| Position | L0.0 A5.4 F4.1 mm |
| Orientation | T > S2.3 > C-1.6 |
| Phase enc. dir | A >> P |
| FoV read | 208 mm |
| FoV phase | 86.5% |
| Slice thickness | 2.0 mm |
| TR | 1230 ms |
| Multi-slice mode | Interleaved |
| Series | Interleaved |
| Concatenations | 1 |
| **Geometry - AutoAlign** | |
| Slab group | 1 |
| Position | L0.0 A5.4 F4.1 mm |
| Orientation | T > S2.3 > C-1.6 |
| Phase enc. dir | A >> P |
| AutoAlign | Head > Brain |
| Initial position | L0.0 A5.4 F4.1 mm |
| L | 0.0 mm |
| A | 5.4 mm |
| F | 4.1 mm |
| Initial rotation | 0.09 deg |
| Initial orientation | T > S |
| T > S | 2.3 |
| > C | -1.6 |
| **Geometry - Saturation** | |
| Fat suppr. | Fat sat. |
| Special sat. | None |
| **System - Misc** | |
| Positioning mode | REF |
| Table position | H |
| Table position | 0 mm |
| MSMA | S - C - T |
| Sagittal | R >> L |
| Coronal | A >> P |
| Transversal | F >> H |
| Coil Combine Mode | Sum of squares |
| Optimisation | Speed |
| AutoAlign | Head > Brain |
| Coil selection | Default |
| **System - Adjustment** | |
| B0 shim mode | Standard |
| B1 shim mode | TrueForm |
| Adjust with body coil | Off |
| Confirm freq. adjustment | Off |
| Assume Dominant Fat | Off |
| Assume Silicone | Off |
| Adjustment Tolerance | Auto |
| **System - Adjustment volume** | |
| Position | L0.0 A5.4 F4.1 mm |
| Orientation | T > S2.3 > C-1.6 |
| Rotation | 0.09 deg |
| A >> P | 180 mm |
| R >> L | 208 mm |
| F >> H | 156 mm |
| Reset | Off |
| **System - pTx Volume** | |
| B1 shim mode | TrueForm |
| Excitation | Standard |
| **System - Tx/Rx** | |
| Frequency 1H | 123.225865 MHz |
| Correction factor | 1 |
| Gain | High |
| Image Scale Cor. | 1.000 |
| Reset | Off |
| ? Ref. Amplitude 1H | 0.000 V |
| **Physio - Signal 1** | |
| 1st Signal/Mode | None |
| TR | 1230 ms |
| Concatenations | 1 |
| **BOLD** | |
| GLM statistics | Off |
| Dynamic t-maps | Off |
| Ignore meas. at start | 0 |
| Ignore after transition | 0 |
| Model transition states | On |
| Temp. highpass filter | On |
| Threshold | 4.00 |
| Paradigm size | 40 (20 baseline, 20 active) |
| Motion correction | Off |
| Spatial filter | Off |
| Measurements | 1000 |
| Delay in TR | 0 ms |
| Multiple series | Off |
| **Sequence - Part 1** | |
| Introduction | On |
| Multi-slice mode | Interleaved |
| Free echo spacing | Off |
| Echo spacing | 0.69 ms |
| Bandwidth | 1780 Hz/Px |
| **Sequence - Part 2** | |
| EPI factor | 90 |
| RF pulse type | Normal |
| Gradient mode | Fast |
| Excitation | Standard |

| **Reference GRE (no SMS)** | |
| --- | --- |
| TA:0.24 PM: FIX Voxel size:2.0x2.0x2.0 mmPAT: Off Rel. SNR:1.00 :epfid | |
| **Properties** | |
| Prio Recon. | Off |
| Load to viewer | On |
| Inline movie | Off |
| Auto store images | On |
| Load to stamp segments | Off |
| Load to graphic segments | Off |
| Auto open inline display | Off |
| Auto close inline display | Off |
| Start measurement without further preparation | Off |
| Wait for user to start | Off |
| Start measurements | Single |
| **Routine** | |
| Slab group | 1 |
| Slabs | 78 |
| Dist. factor | 0% |
| Position | L0.0 A5.4 F4.1 mm |
| Orientation | T > S2.3 > C-1.6 |
| Phase enc. dir | A >> P |
| AutoAlign | Head > Brain |
| Phase oversampling | 0% |
| FoV read | 208 mm |
| FoV phase | 86.5% |
| Slice thickness | 2.0 mm |
| TR | 7530 ms |
| TE | 32.0 ms |
| Averages | 1 |
| Concatenations | 1 |
| Filter | Prescan normalisation |
| Coil elements | HC1-7 |
| **Contrast - general** | |
| TR | 7530 ms |
| TE | 32.0 ms |
| MTC | Off |
| Flip angle | 65 deg |
| Fat suppr. | Fat sat. |
| **Contrast - dynamic** | |
| Averages | 1 |
| Averaging mode | Long term |
| Reconstruction | Magnitude |
| Measurements | 2 |
| Delay in TR | 0 ms |
| Multiple series | Off |
| **Resolution - general** | |
| FoV read | 208 mm |
| FoV phase | 86.5% |
| Slice thickness | 2.0 mm |
| Base resolution | 104 |
| Phase resolution | 100% |
| Phase partial Fourier | 7/8 |
| Interpolation | Off |
| **Resolution - iPAT** | |
| Acceleration mode | None |
| **Resolution - filter image** | |
| Image filter | Off |
| Prescan norm. | On |
| **Resolution - filter raw data** | |
| Raw filter | Off |
| Elliptical filter | Off |
| Hamming | Off |
| **Geometry - general** | |
| Slab group | 1 |
| Slabs | 78 |
| Dist. factor | 0% |
| Position | L0.0 A5.4 F4.1 mm |
| Orientation | T > S2.3 > C-1.6 |
| Phase enc. dir | A >> P |
| FoV read | 208 mm |
| FoV phase | 86.5% |
| Slice thickness | 2.0 mm |
| TR | 7530 ms |
| Multi-slice mode | Interleaved |
| Series | Interleaved |
| Concatenations | 1 |
| **Geometry - AutoAlign** | |
| Slab group | 1 |
| Position | L0.0 A5.4 F4.1 mm |
| Orientation | T > S2.3 > C-1.6 |
| Phase enc. dir | A >> P |
| AutoAlign | Head > Brain |
| Initial position | L0.0 A5.4 F4.1 mm |
| L | 0.0 mm |
| A | 5.4 mm |
| F | 4.1 mm |
| Initial rotation | 0.09 deg |
| Initial orientation | T > S |
| T > S | 2.3 |
| > C | -1.6 |
| **Geometry - Saturation** | |
| Fat suppr. | Fat sat. |
| Special sat. | None |
| **System - Misc** | |
| Positioning mode | FIX |
| Table position | H |
| Table position | 0 mm |
| MSMA | S - C - T |
| Sagittal | R >> L |
| Coronal | A >> P |
| Transversal | F >> H |
| Coil Combine Mode | Sum of squares |
| Optimisation | Speed |
| AutoAlign | Head > Brain |
| Coil selection | Default |
| **System - Adjustment** | |
| B0 shim mode | Standard |
| B1 shim mode | TrueForm |
| Adjust with body coil | Off |
| Confirm freq. adjustment | Off |
| Assume Dominant Fat | Off |
| Assume Silicone | Off |
| Adjustment Tolerance | Auto |
| **System - Adjustment volume** | |
| Position | L0.0 A5.4 F4.1 mm |
| Orientation | T > S2.3 > C-1.6 |
| Rotation | 0.09 deg |
| A >> P | 180 mm |
| R >> L | 208 mm |
| F >> H | 156 mm |
| Reset | Off |
| **System - pTx Volume** | |
| B1 shim mode | TrueForm |
| Excitation | Standard |
| **System - Tx/Rx** | |
| Frequency 1H | 123.225865 MHz |
| Correction factor | 1 |
| Gain | High |
| Image Scale Cor. | 1.000 |
| Reset | Off |
| ? Ref. Amplitude 1H | 0.000 V |
| **Physio - Signal 1** | |
| 1st Signal/Mode | None |
| TR | 7530 ms |
| Concatenations | 1 |
| **BOLD** | |
| GLM statistics | Off |
| Dynamic t-maps | Off |
| Ignore meas. at start | 0 |
| Ignore after transition | 0 |
| Model transition states | On |
| Temp. highpass filter | On |
| Threshold | 4.00 |
| Paradigm size | 40 (20 baseline, 20 active) |
| Motion correction | Off |
| Spatial filter | Off |
| Measurements | 2 |
| Delay in TR | 0 ms |
| Multiple series | Off |
| **Sequence - Part 1** | |
| Introduction | On |
| Multi-slice mode | Interleaved |
| Free echo spacing | Off |
| Echo spacing | 0.69 ms |
| Bandwidth | 1780 Hz/Px |
| **Sequence - Part 2** | |
| EPI factor | 90 |
| RF pulse type | Normal |
| Gradient mode | Fast |
| Excitation | Standard |

| **Reference SE (no SMS; AP & PA)** | |
| --- | --- |
| TA:0:50 PM: FIX Voxel size:2.0x2.0x2.0 mmPAT: Off Rel. SNR:1.00 :epse | |
| **Properties** | |
| Prio Recon. | Off |
| Load to viewer | On |
| Inline movie | Off |
| Auto store images | On |
| Load to stamp segments | Off |
| Load to graphic segments | Off |
| Auto open inline display | Off |
| Auto close inline display | Off |
| Start measurement without further preparation | Off |
| Wait for user to start | Off |
| Start measurements | Single |
| **Routine** | |
| Slab group | 1 |
| Slabs | 78 |
| Dist. factor | 0% |
| Position | L0.0 A5.4 F4.1 mm |
| Orientation | T > S2.3 > C-1.6 |
| Phase enc. dir | A >> P (P >> A) |
| AutoAlign | Head > Brain |
| Phase oversampling | 0% |
| FoV read | 208 mm |
| FoV phase | 86.5% |
| Slice thickness | 2.0 mm |
| TR | 16200 ms |
| TE | 93.0 ms |
| Concatenations | 1 |
| Filter | Raw data, Prescan normalisation |
| Coil elements | HC1-7 |
| **Contrast - general** | |
| TR | 16200 ms |
| TE | 93.0 ms |
| MTC | Off |
| Magn. preparation | None |
| Fat suppr. | Fat sat. |
| Fat suppr. mode | Strong |
| **Contrast - dynamic** | |
| Averaging mode | Long term |
| Reconstruction | Magnitude |
| Measurements | 1 |
| Delay in TR | 0 ms |
| Multiple series | Off |
| **Resolution - general** | |
| FoV read | 208 mm |
| FoV phase | 86.5% |
| Slice thickness | 2.0 mm |
| Base resolution | 104 |
| Phase resolution | 100% |
| Phase partial Fourier | 7/8 |
| Interpolation | Off |
| **Resolution - iPAT** | |
| Acceleration mode | None |
| **Resolution - filter image** | |
| Distortion Corr. | Off |
| Prescan norm. | On |
| Dynamic field corr. | Off |
| **Resolution - filter raw data** | |
| Raw filter | On |
| Elliptical filter | Off |
| **Geometry - general** | |
| Slab group | 1 |
| Slabs | 78 |
| Dist. factor | 0% |
| Position | L0.0 A5.4 F4.1 mm |
| Orientation | T > S2.3 > C-1.6 |
| Phase enc. dir | A >> P |
| FoV read | 208 mm |
| FoV phase | 86.5% |
| Slice thickness | 2.0 mm |
| TR | 16200 ms |
| Multi-slice mode | Interleaved |
| Series | Interleaved |
| Concatenations | 1 |
| **Geometry - AutoAlign** | |
| Slab group | 1 |
| Position | L0.0 A5.4 F4.1 mm |
| Orientation | T > S2.3 > C-1.6 |
| Phase enc. dir | A >> P (P >> A) |
| AutoAlign | Head > Brain |
| Initial position | L0.0 A5.4 F4.1 mm |
| L | 0 mm |
| A | 5.4 mm |
| F | 4.1 mm |
| Initial rotation | 0.09 deg (-179.91 deg) |
| Initial orientation | T > S |
| T > S | 2.3 |
| > C | -1.6 |
| **Geometry - Saturation** | |
| Fat suppr. | Fat sat. |
| Fat suppr. mode | Strong |
| Special sat. | None |
| **System - Misc** | |
| Positioning mode | FIX |
| Table position | H |
| Table position | 0 mm |
| MSMA | S - C - T |
| Sagittal | R >> L |
| Coronal | A >> P |
| Transversal | F >> H |
| Coil Combine Mode | Adaptative combine |
| Optimisation | Off |
| AutoAlign | Head > Brain |
| Coil selection | Default |
| **System - Adjustment** | |
| B0 shim mode | Standard |
| B1 shim mode | TrueForm |
| Adjust with body coil | Off |
| Confirm freq. adjustment | Off |
| Assume Dominant Fat | Off |
| Assume Silicone | Off |
| Adjustment Tolerance | Auto |
| **System - Adjustment volume** | |
| Position | L0.0 A5.4 F4.1 mm |
| Orientation | T > S2.3 > C-1.6 |
| Rotation | 0.09 deg (-179.91 deg) |
| A >> P | 180 mm |
| R >> L | 208 mm |
| F >> H | 156 mm |
| Reset | Off |
| **System - pTx Volume** | |
| B1 shim mode | TrueForm |
| Excitation | Standard |
| **System - Tx/Rx** | |
| Frequency 1H | 123.225865 MHz |
| Correction factor | 1 |
| Gain | High |
| Image Scale Cor. | 1.000 |
| Reset | Off |
| ? Ref. Amplitude 1H | 0.000 V |
| **Physio - Signal 1** | |
| 1st Signal/Mode | None |
| TR | 16200 ms |
| Concatenations | 1 |
| **Physio – PACE** |  |
| Rsp. control | Off |
| Concatenations | 1 |
| **Diff – Neuro** |  |
| Diffusion mode | Free |
| Diff. directions | 6 |
| Diff. scheme | Bipolar |
| Diff. weightings | 1 |
| b-value | 0 s/mm² |
| b-value | 2 |
| Diff. weighted images | On |
| Trace weighted images | Off |
| ADC maps | Off |
| FA maps | Off |
| Mosaic | Off |
| Tensor | Off |
| Noise level | 40 |
| **Diff – Body** |  |
| Diffusion mode | Free |
| Diff. directions | 6 |
| Diff. scheme | Bipolar |
| Diff. weightings | 1 |
| b-value | 0 s/mm² |
| b-value | 2 |
| Diff. weighted images | On |
| Trace weighted images | Off |
| ADC maps | Off |
| Exponential ADC maps | Off |
| FA maps | Off |
| Invert grey scale | Off |
| Calculated image | Off |
| b-value >= | 0 s/mm² |
| Noise level | 40 |
| **Diff – Composing** |  |
| Distortion corr. | Off |
| **Sequence - Part 1** | |
| Introduction | On |
| Optimisation | None |
| Multi-slice mode | Interleaved |
| Free echo spacing | On |
| Echo spacing | 0.69 ms |
| Bandwidth | 1780 Hz/Px |
| **Sequence - Part 2** | |
| EPI factor | 90 |
| RF pulse type | Normal |
| Gradient mode | Fast |
| Excitation | Standard |

| **T1** | |
| --- | --- |
| TA: 5:12 PM: FIX Voxel size: 1.1×1.1×1.2 mmPAT: 2 Rel. SNR: 1.00 : tfl | |
| **Properties** | |
| Prio Recon. | Off |
| Load to viewer | On |
| Inline movie | Off |
| Auto store images | On |
| Load to stamp segments | Off |
| Load to graphic segments | Off |
| Auto open inline display | Off |
| Auto close inline display | Off |
| Start measurement without further preparation | Off |
| Wait for user to start | On |
| Start measurements | Single |
| **Routine** | |
| Slab group | 1 |
| Slabs | 1 |
| Dist. factor | 50% |
| Position | Isocentre |
| Orientation | Sagittal |
| Phase enc. dir | A >> P |
| AutoAlign | Head > Brain |
| Phase oversampling | 0% |
| Slice oversampling | 0.0% |
| Slices per slab | 176 |
| FoV read | 270 mm |
| FoV phase | 93.8% |
| Slice thickness | 1.2 mm |
| TR | 2300.0 ms |
| TE | 2.95 ms |
| Averages | 1 |
| Concatenations | 1 |
| Filter | Normalisation |
| Coil elements | HC1-7; NC1,2 |
| **Contrast - general** | |
| TR | 2300.0 ms |
| TE | 2.95 ms |
| Magn. preparation | Non-sel. IR |
| TI | 900 ms |
| Flip angle | 9 deg |
| Fat suppr. | None |
| Water suppr. | None |
| **Contrast - dynamic** | |
| Averages | 1 |
| Averaging mode | Long term |
| Reconstruction | Magnitude |
| Measurements | 1 |
| Multiple series | Off |
| **Resolution - general** | |
| FoV read | 270 mm |
| FoV phase | 93.8% |
| Slice thickness | 1.2 mm |
| Base resolution | 256 |
| Phase resolution | 100% |
| Slice resolution | 100% |
| Phase partial Fourier | Off |
| Slice partial Fourier | Off |
| Interpolation | Off |
| **Resolution - iPAT** | |
| PAT mode | GRAPPA |
| Accel. factor PE | 2 |
| Ref. lines PE | 32 |
| Accel. factor 3D | 1 |
| Reference scan mode | Integrated |
| **Resolution - filter image** | |
| Image filter | Off |
| Distortion corr. | Off |
| Prescan norm. | Off |
| Normalize | On |
| B1 filter | Off |
| **Resolution - filter raw data** | |
| Raw filter | Off |
| Elliptical filter | Off |
| **Geometry - general** | |
| Slab group | 1 |
| Slabs | 1 |
| Dist. factor | 50% |
| Position | Isocentre |
| Orientation | Sagittal |
| Phase enc. dir | A >> P |
| AutoAlign | Head > Brain |
| Slice oversampling | 0.0% |
| Slices per slab | 176 |
| FoV read | 270 mm |
| FoV phase | 93.8% |
| Slice thickness | 1.2 mm |
| TR | 2300.0 ms |
| Multi-slice mode | Single shot |
| Series | Interleaved |
| Concatenations | 1 |
| **Geometry - AutoAlign** | |
| Slab group | 1 |
| Position | Isocentre |
| Orientation | Sagittal |
| Phase enc. dir | A >> P |
| Initial position | Isocentre |
| L | 0.0 mm |
| P | 0.0 mm |
| H | 0.0 mm |
| Initial rotation | 0.00 deg |
| Initial orientation | Transversal |
| **System - Misc** | |
| Positioning mode | FIX |
| Table position | H |
| Table position | 0 mm |
| MSMA | S - C - T |
| Sagittal | R >> L |
| Coronal | A >> P |
| Transversal | F >> H |
| Coil Combine Mode | Adaptative Combine |
| Save uncombined | Off |
| Optimisation | Off |
| AutoAlign | Head > Brain |
| Coil selection | Off - AutoCoilSelect |
| **System - Adjustment** | |
| B0 shim mode | Standard |
| B1 shim mode | TrueForm |
| Adjust with body coil | Off |
| Confirm freq. adjustment | Off |
| Assume Dominant Fat | Off |
| Assume Silicone | Off |
| Adjustment Tolerance | Auto |
| **System - Adjustment volume** | |
| Position | Isocentre |
| Orientation | Sagittal |
| Rotation | 0.00 deg |
| A >> P | 254 mm |
| R >> L | 270 mm |
| F >> H | 212 mm |
| Reset | Off |
| **System - pTx Volume** | |
| B1 shim mode | TrueForm |
| Excitation | Non-sel |
| **System - Tx/Rx** | |
| Frequency 1H | 123.225865 MHz |
| Correction factor | 1 |
| Gain | Low |
| Image Scale Cor. | 1.000 |
| Reset | Off |
| ? Ref. Amplitude 1H | 0.000 V |
| **Physio - Signal 1** | |
| 1st Signal/Mode | Non-sel IR |
| TR | 2400 ms |
| Concatenations | 1 |
| **Physio - Heart** | |
| Magn. preparation | Non-sel. IR |
| TI | 900 ms |
| Fat suppr. | None |
| Dark blood | Off |
| FoV read | 270 mm |
| FoV phase | 93.8% |
| Phase resolution | 100% |
| **Physio - PACE** | |
| Resp. control | Off |
| Concatenations | 1 |
| **Inline - General** | |
| Subtraction | Off |
| Measurements | 1 |
| StdDev | Off |
| Save original images | On |
| **Inline - MIP** | |
| MIP-Sag | Off |
| MIP-Cor | Off |
| MIP-Tra | Off |
| MIP-Time | Off |
| Save original images | On |
| **Inline - Composing** | |
| Distortion corr. | Off |
| **Sequence - Part 1** | |
| Introduction | On |
| Dimension | 3D |
| Elliptical scanning | Off |
| Reordering | Linear |
| Asymetric echo | Off |
| Flow comp. | No |
| Multi-slice mode | Single shot |
| Echo spacing | 7 ms |
| Bandwidth | 240 Hz/Px |
| **Sequence - Part 2** | |
| RF pulse type | Fast |
| Gradient mode | Normal |
| Excitation | Non-sel |
| RF spoiling | On |
| Mode | Off |
| Turbo factor | 176 |
| **Sequence - Assistence** | |
| Mode | Off |
